# Poisoning by Chloroform

**Published:** 1862-07

**Authors:** 


Poisoning by Chloroform.—Dr. W. Marcet offers some practical observations on this subject. When chloroform is inhaled, it passes rapidly into the blood, and thence to the brain. The administration being suspended, the chloroform is eliminated from the body by the respiration, each inspiration displacing most of the vapor contained in the blood exposed by the lungs to the action of air during that inspiration. If the air inspired be pure, the displacement of chloroform will be great; if the air contains chloroform, the displacement will be less. When a patient begins to inhale chloroform, a portion is absorbed by the blood, the remainder expired; but shortly afterwards, in addition to the expiration of that part of the chloroform which has not been taken up by the blood, a certain quantity of that which has been absorbed is also ejected, being displaced by the air mixed with the chloroform inhaled. At this stage, however, there is still an accumulation of the anæsthetic agent in the blood, more being taken into the circulation than given out; gradually complete insensibility is produced. As soon as the handkerchief is removed, the patient begins ridding himself rapidly of the chloroform, and recovers consciousness, unless more of the anæsthetic agent be exhibited. When the state of insensibility is thus kept up, the accumulation of the vapor in the blood obviously takes place no longer, otherwise it would invariably produce death; there must consequently be an equilibrium between the quantity of chloroform absorbed and that which is eliminated by respiration. If, during this stage of insensibility, from any cause whatever, the power of absorption of the blood for chloroform be suddenly increased, or its property of giving it out to the air inspired be diminished, then death will take place from an accumulation of the vapor in the blood. The absorbing power of the blood can not well be increased, but elimination may be impaired by the administration of the chloroform vapor in too concentrated a condition. An excess of chloroform in the air prevents or interferes with the exit of that already existing in the blood ; while the blood goes on taking up chloroform, it gives out less than a quantity equal to that absorbed ; at the same time the evil may be increased by a few deep inspirations taken unconsciously, although apparently with the view of ejecting the poison, and life is suddenly extinguished. In drunkards, the delicate membrane of the air-cells becomes thickened, and this acts at first more or less as an obstacle to the admission of chloroform into the blood, while it afterwards again checks the elimination of the vapor absorbed ; the result is an excessive accumulation of chloroform in the blood.

Conclusions : (*a.*) Chloroform must be administered cautiously, and its effects watched with particular attention, if although the vapor be freely inhaled, the patient does not become insensible within the usual time.

(*b.*) As soon as the state of insensibility is obtained, the vapor must be exhibited diluted as much as possible with pure air, and air free from the anæsthetic agent ought to be allowed frequently into the lungs.

(*c.*) Great attenlion should be paid to the state of the respiration, which ought to guide the exhibition of the anæsthetic agent still more than the condition of the pulse. If the inspirations become less deep and respiration appears failing, air free from chloroform ought to be immediately allowed into the lungs.

(*d.*) When a patient has sunk under the effects of chloroform, the only means of restoring animation is by artificial respiration, introducing as much air as possible into the lungs, and at the same lime stimulating the action of the heart.—*Med. Times and Gazette.*

